# The role of multimorbidity in short-term mortality of lung cancer patients in Spain: a population-based cohort study

**DOI:** 10.1186/s12885-021-08801-9

**Published:** 2021-09-24

**Authors:** Maja Niksic, Daniel Redondo-Sanchez, Yoe-Ling Chang, Miguel Rodriguez-Barranco, Jose Exposito-Hernandez, Rafael Marcos-Gragera, Ester Oliva-Poch, Joaquim Bosch-Barrera, Maria-Jose Sanchez, Miguel Angel Luque-Fernandez

**Affiliations:** 1 Department of Non-Communicable Disease Epidemiology, Inequalities in Cancer Outcomes Network, London School of Hygiene and Tropical Medicine, London, UK; 2 Non-Communicable Disease and Cancer Epidemiology Group, Instituto de Investigación Biosanitaria de Granada (ibs. GRANADA), Granada, Spain; 3 Biomedical Network Research Centers of Epidemiology and Public Health (CIBERESP), Madrid, Spain; 4 Andalusian School of Public Health (EASP), Granada Cancer Registry, Granada, Spain; 5 Department of Oncology, HU Virgen de las Nieves, Granada, Spain; 6 Research Group on Statistics, Econometrics and Health (GRECS), University of Girona, Girona, Spain; 7 Department of Medical Oncology, Institut Català d’Oncologia Hospital Universitari de Girona Dr. Josep Trueta, Girona, Spain; 8 Descriptive Epidemiology, Genetics and Cancer Prevention Group, Biomedical Research Institute (IDIBGI), Girona, Spain; 9 Epidemiology Unit and Girona Cancer Registry, Oncology Coordination Plan, Catalan Institute of Oncology, Girona, Spain; 10 Radiation Oncology Department, Catalan Institute of Oncology, Hospital Trueta, Girona, Spain; 11 Department of Public Health and Preventive Medicine, University of Granada, Granada, Spain

**Keywords:** Lung neoplasms, Comorbidity, Multimorbidity, Mortality, cancer epidemiology

## Abstract

**Aim:**

Chronic diseases often occur simultaneously and tend to be associated with adverse health outcomes, but limited research has been undertaken to understand their role in lung cancer mortality. Therefore, this study aims to describe the prevalence and patterns of having one (comorbidity) or ≥ 2 chronic diseases (multimorbidity) among lung cancer patients in Spain, and to examine the association between comorbidity or multimorbidity and short-term mortality risk at six months after cancer diagnosis.

**Methods:**

In this population-based cohort study, data were drawn from two Spanish population-based cancer registries, Girona and Granada, and electronic health records. We identified 1259 adult lung cancer patients, diagnosed from 1st January 2011 to 31st December 2012. We identified the most common patterns of individual comorbidities and their pairwise correlations. We used a flexible parametric modelling approach to assess the overall short-term mortality risk 6 months after cancer diagnosis by levels of comorbidity after adjusting for age, sex, smoking status, province of residence, surgery, cancer stage, histology, and body mass index.

**Results:**

We found high prevalence of comorbidity in lung cancer patients, especially among the elderly, men, those diagnosed with advanced-stage tumours, smokers, and obese patients. The most frequent comorbidities were chronic obstructive pulmonary disease (36.6%), diabetes (20.7%) and heart failure (16.8%). The strongest pairwise correlation was the combination of heart failure with renal disease (r = 0.20, *p* < 0.01), and heart failure with diabetes (r = 0.16, p < 0.01). Patients with either one or two or more comorbidities had 40% higher overall mortality risk than those without comorbidities (aHR for comorbidity: 1.4, 95%CI: 1.1–1.7; aHR for multimorbidity: 1.4, 95%CI: 1.1–1.8), when relevant confounding factors were considered.

**Conclusions:**

The presence of comorbid diseases, rather than the number of comorbidities, was associated with increasing the risk of short-term lung cancer mortality in Spain. Comorbidity was a consistent and independent predictor of mortality among lung cancer patients, six months after diagnosis. The most common comorbid conditions were age-, obesity- and tobacco-related diseases. Our findings highlight the need to develop targeted preventive interventions and more personalised clinical guidelines to address the needs of lung cancer patients with one or more comorbidities in Spain.

**Supplementary Information:**

The online version contains supplementary material available at 10.1186/s12885-021-08801-9.

## Background

Lung cancer is the most commonly diagnosed cancer worldwide and the leading cause of cancer death, accounting for 18.4% of the total cancer deaths in both men and women combined [1]. During 2020 the estimated number of new cases in Spain was 29,188 and 22,930 estimated deaths, with the European age-standardised mortality rate of 47.6 per 100,000 [2]. There is an increasing trend in lung cancer incidence and mortality among women [3]. Over the last decade, Spain had the second highest average increase in lung cancer incidence among women after Brazil; and the largest increase in lung cancer mortality than any other country in the world [4].

Poor prognosis and increased mortality among lung cancer patients are often associated with advanced age and stage at diagnosis, identified in half of all patients [5, 6]. The elderly are more likely to be diagnosed with lung cancer and to experience comorbidity (coexistence of an additional chronic disease) or multimorbidity (coexistence of more than two chronic diseases) with a primary condition [7, 8]. Multimorbidity is more common among lung cancer patients in comparison with patients diagnosed with other types of cancer, such as breast, prostate or colorectal cancer [9, 10]. However, the effects of multimorbidity on lung cancer mortality have not been adequately examined and available evidence is conflicting. Comorbidity and multimorbidity were independent prognostic factors for lung cancer patients, significantly related to an increasing mortality risk in some studies, [11] but not in others [12–15]. Most of the previous studies did not account for relevant lifestyle and behavioural confounders, such as obesity or smoking status, [16, 17] and none of them were conducted in Spain. This study has two purposes: first, to describe the prevalence of comorbidities and multimorbidity and their pattern of pairwise correlations in lung cancer patients in Spain and, second, to examine the association between multimorbidity and short-term mortality at 6 months following the diagnosis of lung cancer, adjusting for relevant clinical and lifestyle confounders.

## Materials and methods

This population-based cohort study included all primary lung and bronchus cancer incident cases diagnosed from 1st January 2011 to 31st December 2012 in two Spanish population-based cancer registries - Girona and Granada. The eligibility criteria included all adult patients (aged 18 and over) diagnosed with primary lung and bronchus malignant cancer, during 2011 and 2012 in Girona or Granada. We excluded patients younger than 18 years or those diagnosed with a secondary lung cancer. The entry date was defined as the date of cancer diagnosis, while exit date was defined as the date of death or the date at 6 months after their cancer diagnosis, whichever occurred first.

The data collection followed a detailed protocol from the European High Resolution studies collaboration (TRANSCAN-HIGHCARE project within the ERA-Net) [13]. Lung cancer types and histological codes were classified according to the International Classification of Diseases for Oncology, third edition (ICD-O-3) [18]. Lung cancer types were registered with codes C34.0, C34.1, C34.2, C34.3, C34.8 and C34.9. The histological subgroups were classified as 1) adenocarcinomas, 2) small cell lung cancers, 3) squamous carcinomas, and 4) unspecified and other subgroups (Supplementary Table 1).

We recorded information on patients’ age, sex, smoking status, province of residence, surgery, body mass index (BMI), cancer stage at diagnosis (TNM staging system, 7th edition), tumour histology, comorbidities, and vital status.

Vital status was assessed at 6 months after cancer diagnosis and was ascertained based on information from clinical records linked to the national death registry of the Spanish National Statistics Institute. Short-term mortality at six months was the outcome, and patients’ baseline comorbidity status at cancer diagnosis was the main exposure. A priori confounders were age and sex. Other relevant confounders were province of residence, smoking status, performed surgery, stage at diagnosis, tumour histology and BMI. Province of residence was considered as a proxy measure for socio-economic deprivation. Based on national figures of the household average income from the National Institute of Statistics, Girona in the North of the country is more affluent than Granada in Southern Spain [19]. BMI in kg/m2 was categorised as healthy weight (< 24.9), overweight (25–29.9), and obese (≥30 kg/m2) [20]. Comorbidities were assessed retrospectively six months before cancer diagnosis using all the available information from patients’ electronic health records in primary care, outpatient, and in-patient hospital information. Then, comorbidities were identified using an algorithm based on the codes from the International Classification of Diseases, 10th Revision, and classified based on the Royal College of Surgeons-modified Charlson score (RCS). The RCS reduces the number of comorbidities to 12, removes a category (peptic ulcer disease), and groups diseases (e.g., diabetes mellitus codes with or without complications) (Supplementary Table 2).

In this study comorbidity is defined as the occurrence of a single medical conditions additional to an index disease, i.e. lung cancer [21]. Multimorbidity is defined as the co-occurrence of multiple medical conditions (two or more) in addition to the lung cancer diagnosis (the index disease) [21]. Therefore, the final comorbidity score represents a simple count of the total number of comorbidities for each patient, without assigning any weights, namely: no comorbidities (0), one comorbidity (1), and two or more comorbidities, with (≥2) comorbidities defined as multimorbidity [22].

The study proposal (CP17/00206) was approved by the internal review board of the Andalusian School of Public Health and the ethics committee from the Department of Health of the Andalusian Regional Government (study 0072-N-18). The data are held by the Regional Government of Andalusia and the Andalusian Health Department. This entire study and the research protocol for involving human data was in accordance with the guidelines of the Declaration of Helsinki.

### Statistical analysis

Percentages were used to describe categorical variables, and means and standard deviations for continuous variables. We described the ranked frequency of comorbidities and computed the pairwise correlation (i.e., between pairs of the most common comorbidities) using the Pearson correlation coefficient. In this way, we assessed whether any of the medical conditions that lung cancer patients are experiencing are correlated to one another, and if so, how strong these correlations were. To assess the association between short-term mortality and potential risk factors, in univariate analysis, we computed the number of deaths and person-month at risk, the rates and rates ratios at 6 months post-diagnosis and assessed the presence of linear trends across levels of comorbidities, age, and BMI using the score test for trend. We plotted the short-term cumulative incidence of death at 6 months by comorbidity and multimorbidity status, based on the Aalen-Nelson estimator and assessed statistical significance using the Log-rank test.

In multivariate analysis, to assess the risk of short-term mortality by comorbidity status (including a single comorbidity and multimorbidity), adjusted for relevant confounders, we developed a survival analysis using a flexible parametric modelling approach. The approach allows for a better fit of the baseline hazard, implemented through the smooth transformation of the baseline hazard, using restricted cubic splines with two internal knots and three degrees of freedom [23]. For categorical variables we used the category at lower risk in univariate analysis as a reference. We fitted seven different models including the variables one at each step to assess confounding. From each model we derived the hazard ratios (HR) and 95% confidence intervals (CI). We performed a complete case analysis assuming a pattern of completely missing at random.

To check the consistency of our main results against the missing at random assumption, in sensitivity analysis, we developed a multiple imputation, using chained equations based on a fully conditional specification [24]. We imputed BMI, tumour stage, performed surgery, and smoking status, generating 50 imputed datasets. The results were combined using Rubin’s rules [25]. The model specification for the multiple imputation included the following variables: comorbidities, smoking status, province, BMI, age at diagnosis, sex, tumour stage and histology; and, as auxiliary variables, the Nelson-Aalen cumulative hazard. Furthermore, we assessed the dose-response effect of the number of comorbidities (i.e., 0, 1, 2, and + 3) among lung cancer patients on short-term cancer mortality.

Data were analysed using Stata v.16.1 (StataCorp, College Station, Texas, U.S.) [26].

## Results

A total of 1259 lung cancer cases (83.4% male) were included in the analysis (Supplementary Table 3). Average age was 68.4 years (SD: 11.7). Over half of males (51.9%) and a third of females (35.4%) were above 70 years old. The majority were ever-smokers (87.1%), with 41.3% current smokers and 45.7% former smokers. More than half of patients (58.0%) were above the healthy weight (average BMI: 26.3 kg/m^2^, SD: 4.8). Surgery was performed in 16.6% of the cases. Over half of patients were diagnosed with metastatic tumours (54.7%), with more metastatic tumours diagnosed in Granada (57.8%) than Girona (49.9%, *p* < 0.01). A third of patients (33.4%) had multimorbidity and 28.0% a single comorbidity.

Half of 80+ year-olds had multimorbidity, but 58.9% of below 60-year-olds had no comorbidities (*p* < 0.001, Table 1). Males had higher multimorbidity prevalence than females (35.7% vs. 22.0%, respectively *p* < 0.001). Former smokers had the highest multimorbidity prevalence (44.4% vs. 26.9% never-smokers, *p* < 0.001). Obese patients had double prevalence figures than those with healthy weight (48.3% vs. 24.5%, respectively p < 0.001). A third of patients with squamous carcinoma, and” Other” histology subgroup, including unspecified carcinomas, had multimorbidity (36.5% vs. 38.4%, p < 0.001).

**Table 1:** Vital status at six-months, sociodemographic characteristics, smoking status, province of residence, body mass index, cancer surgery, histology, and TNM stage by multimorbidity status among lung cancer patients diagnosed between 2011 and 2012, in two population-based Spanish cancer registries: Girona and Granada **(***n* = 1259 lung cancer patients and 581 deaths at six-months after cancer diagnosis)

Variable	Royal College of Surgeons-modified Charlson score			
	No comorbidity	One comorbidity	Two or more comorbidities	***p***-value
	N (%)	N (%)	N (%)	
**Vital status at 6 months**				0.001
Alive	290 (42.8)	189 (27.9)	199 (29.4)	
Dead	196 (33.7)	164 (28.2)	221 (38.0)	
**Age at diagnosis, years**				< 0.001
< 60	179 (58.9)	77 (25.3)	48 (15.8)	
60–69	132 (39.4)	111 (33.1)	92 (27.5)	
70–79	110 (28.4)	113 (29.2)	164 (42.4)	
≥ 80	65 (27.9)	52 (22.3)	116 (49.8)	
**Sex**				< 0.001
Male	355 (33.8)	321 (30.6)	374 (35.6)	
Female	131 (62.7)	32 (15.3)	46 (22.0)	
**Smoking status**				< 0.001
Current smoker	187 (41.3)	144 (31.8)	122 (26.9)	
Previous smoker	126 (25.2)	153 (30.5)	222 (44.3)	
Never smoked	65 (45.8)	37 (26.1)	40 (28.2)	
**Province**				0.019
Girona	216 (43.3)	133 (26.7)	150 (30.1)	
Granada	270 (35.5)	220 (29.0)	270 (35.5)	
**Body Mass Index (kg/m**^**2**^**)**				< 0.001
Healthy weight (< 25.0)	142 (45.8)	92 (29.7)	76 (24.5)	
Overweight (25.0–29.9)	99 (35.9)	90 (32.6)	87 (31.5)	
Obese (≥30)	33 (21.9)	45 (29.8)	73 (48.3)	
**Surgery**				0.163
Yes	81 (40.1)	63 (31.2)	58 (28.7)	
No	362 (35.8)	289 (28.6)	361 (35.7)	
**Histology**				< 0.001
ADE	176 (46.1)	101 (26.4)	105 (27.5)	
Others	134 (35.5)	99 (26.2)	145 (38.4)	
SCLC	73 (44.2)	44 (26.7)	48 (29.1)	
SQUA	103 (30.8)	109 (32.6)	122 (36.5)	
**TNM stage**				0.014
I	0 (0.0)	1 (20.0)	4 (80.0)	
II	67 (35.1)	61 (31.9)	63 (33.0)	
III	107 (30.7)	106 (30.4)	136 (39.0)	
IV	267 (40.5)	183 (27.8)	209 (31.7)	

**Histology: ADE**: adenocarcinoma; **SCLC**: small cell lung cancer; **SQUA**: Squamous carcinoma;

**Others**: non-small cell lung cancer, large cell lung cancer, neuroendocrine lung cancer,

and unspecified lung cancer. *P*-values based on Chi-square tests

**Missing values:** BMI n (%) = 522 (41.5), Performance status n (%) = 229 (18.2),

Smoking status n (%) = 163 (13.0), TNM stage n (%) = 55 (4.4), Surgery n (%) = 45 (3.6)

**Fig. 1:**
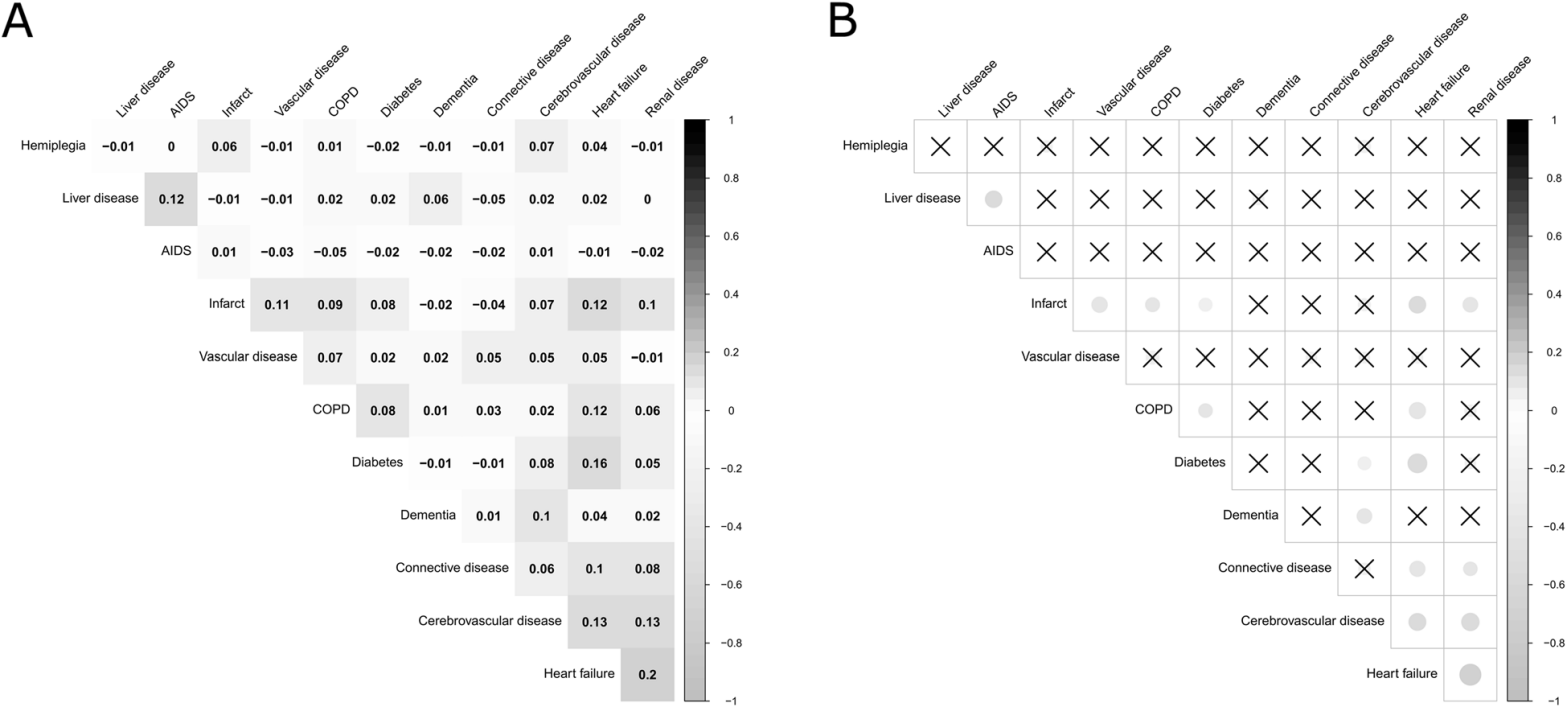
Correlogram of comorbidities among Lung cancer patients in Granada and Girona, Spain, diagnosed during 2011–2012 (*n* = 1260). **A)** Pearson’s correlation coefficient for the pairwise correlations between the most common comorbidities; **B)** Pearson’s correlation two-side significance value set at p < 0.01 for the pairwise correlations between the most common comorbidities

The most prevalent comorbidities were chronic obstructive pulmonary disease (COPD) (36.6%), diabetes (20.7%) and heart failure (16.8%). The most frequent pairwise combination was COPD and diabetes (8.7%), followed by COPD and heart failure (7.9%) (Supplementary Table 4). Figure 1 shows the pattern of pairwise correlations between the most common comorbidities (Fig. 1A) and the two-side significance value set at 0.01 (Fig. 1B). The highest pairwise correlation was between heart failure and renal disease (r = 0.20, *p* < 0.01), followed by heart failure and diabetes (r = 0.16, p < 0.01).

**Fig. 2:**
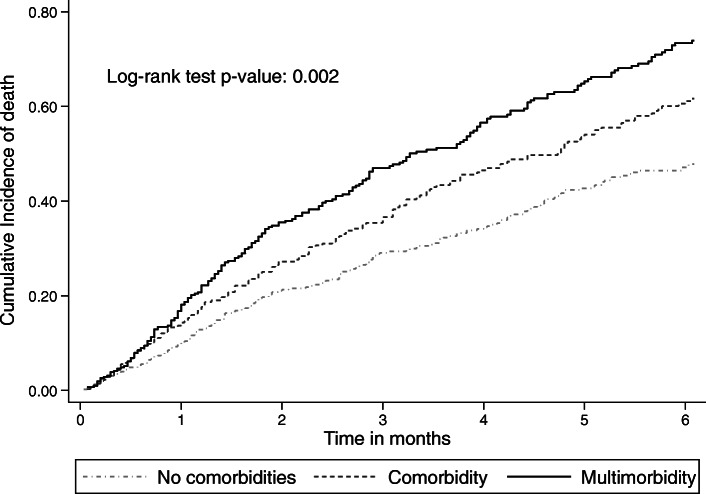
Cumulative probability of death during the first 6 months after lung cancer diagnosis by comorbidity status

Among 5391 person-month at risk, 581 people died before six-months of follow-up i.e., 196 patients without comorbidity, 164 with a single comorbidity, and 228 with multimorbidity (Table 2). The overall observed short-term mortality rate was 10.8 per 100 person-month at risk (95%CI: 9.9–11.7). Figure 2 shows that the cumulative incidence of death at six-months was increasing across the two levels of comorbidity, with the highest risk among those with multimorbidity (log-rank test *p* = 0.002). In univariate analysis, patients with multimorbidity also had the highest overall short-term mortality rate (i.e., MR: 13.0; 95% CI: 11.4–14.8 per 100 person-month) and mortality rate ratio (i.e., MRR: 1.4; 95% CI: 1.2–1.7) compared with patients with one or no comorbidity.

**Table 2:** Short–term (six–months) mortality rates and rates ratios by comorbidity status, sex, age, smoking status, province of residence, BMI, surgery, cancer histology, and TNM stage among lung cancer patients diagnosed between 2010 and 2012, in two population-based Spanish cancer registries: Girona and Granada (*n* = 1259 lung cancer patients and 581 deaths at six–month after cancer diagnosis)

Variables	Deaths / PM	MR per 100 PM (95% CI)	MRR (95% CI)	P–value
**RCS-modified Charlson score**				< 0.001*
No comorbidity	196 / 2166	9.0 (7.9–10.4)	Ref.	
One comorbidity	164 / 1526	10.7 (9.2–12.5)	1.2 (1.0–1.5)	
Two or more comorbidities	221 / 1698	13.0 (11.4–14.8)	1.4 (1.2–1.7)	
**Sex**				0.009
Male	502 / 4435	11.3 (10.4–12.4)	Ref.	
Female	79 / 956	8.3 (6.6–10.3)	1.4 (1.1–1.7)	
**Age at diagnosis (years)**				< 0.001*
< 60	108 / 1470	7.3 (6.1–8.9)	Ref.	
60–69	131 / 1569	8.3 (7.0–9.9)	1.1 (0.9–1.5)	
70–79	181 / 1630	11.1 (9.6–12.8)	1.5 (1.2–1.9)	
≥ 80	161 / 721	22.3 (19.1–26.1)	3.0 (2.4–3.9)	
**Smoking status at diagnosis**				0.176
Never smoked	53 / 687	7.7 (5.9–10.1)	Ref.	
Previous smoker	222 / 2199	10.1 (8.9–11.5)	1.3 (1.0–1.8)	
Current smoker	204 / 2002	10.2 (8.9–11.7)	1.3 (1.0–1.8)	
**Province**				< 0.001
Girona	189 / 2274	8.3 (7.2–9.6)	Ref.	
Granada	392 / 3116	12.6 (11.4–13.9)	1.5 (1.3–1.8)	
**Body Mass Index (kg/m**^**2**^**)**				0.101*
Healthy weight (< 25.0)	96 / 1551	6.2 (5.1–7.6)	Ref.	
Overweight (25.0–29.9)	82 / 1427	5.7 (4.6–7.1)	0.9 (0.7–1.2)	
Obese (≥30)	40 / 796	5.0 (3.7–6.9)	0.8 (0.6–1.2)	
**Surgery**				< 0.001
Yes	8 / 1216	0.7 (0.3–1.3)	Ref.	
No	541 / 4084	13.2 (12.2–14.4)	20.1 (10.0–40.5)	
**Histology**				0.002
ADE	152 / 1800	8.4 (7.2–9.9)	Ref.	
Others	247 / 1215	20.3 (17.9–23.0)	2.4 (2.0–2.9)	
SCLC	66 / 715	9.2 (7.3–11.8)	1.1 (0.8–1.5)	
SQUA	116 / 1654	7.0 (5.8–8.4)	0.8 (0.7–1.1)	
**TNM stage**				< 0.001
I–III (No-metastasis)	143 / 2810	5.1 (4.3–6.0)	Ref.	
IV (Metastasis)	398 / 2474	16.1 (14.6–17.8)	3.2 (2.6–3.8)	

* Test for trend p-value

**CI:** Confidence Interval; **MR:** Mortality Rate**; MRR:** Mortality Rate Ratio**; PM:** Person–month**;**

**Histology**: **ADE**: adenocarcinoma; **SCLC**: small cell lung cancer; **SQUA**: Squamous carcinoma; **Others**: non-small cell lung cancer, large cell lung cancer, neuroendocrine lung cancer, and unspecified lung cancer

Results from the multiple imputation model (Table 3: model 8) were consistent with the complete case analysis (Table 3: model 7). After adjusting for all confounders in model 7, patients with comorbidity or multimorbidity showed a 40 and 50% increased short-term mortality risk in comparison with patients without comorbidity (adjusted Hazard Ratio [aHR]: 1.4 and 95% CI: 1.0–2.0 and 1.1–2.2, respectively). Not having surgery increased the mortality risk six times (aHR: 6.2; 95% CI: 3.0–13.1). Patients with metastatic cancer had over two times higher mortality than non-metastatic patients (aHR: 2.5; 95% CI: 1.8–3.5). Patients over 80 years old had two times higher mortality risk than those below 60 years of age (aHR: 2.1; 95% CI: 1.3–3.4). Current smokers had 50% higher mortality risk than never-smokers (aHR: 2.1; 95% CI: 1.1–3.9). Patients from Granada had higher mortality risk than those from Girona (aHR: 1.8; 95% CI: 1.4–2.4). In sensitivity analysis the number of comorbidities did not show a dose-response effect, i.e., when we analysed lung cancer patients with a single comorbidity, two comorbid medical conditions, or three and more comorbid conditions (supplementary Table 5).

**Table 3:** Short-term (six-months) comorbidity and multimorbidity mortality risk adjusted for sex, age, province of residence, smoking status, cancer surgery, histology, TNM stage, and BMI among lung cancer patients in Girona and Granada, Spain in 2011 (n = 1259 lung cancer patients and 581 deaths at six-months after cancer diagnosis)

Variables	Model 1 HR (95%CI)	Model 2 HR (95%CI)	Model 3 HR (95%CI)	Model 4 HR (95%CI)	Model 5 HR (95%CI)	Model 6 HR (95%CI)	Model 7 HR (95%CI)	Model 8 HR (95%CI)
**N (sample size)**	1259	1259	1259	1096	1092	1084	706	1259
**RCS-modified Charlson score**								
No comorbidity	Ref.	Ref.	Ref.	Ref.	Ref.	Ref.	Ref.	Ref.
One comorbidity	1.1 (0.9–1.3)	1.1 (0.9–1.4)	1.1 (0.9–1.4)	1.4 (1.1–1.7)	1.4 (1.1–1.8)	1.5 (1.2–1.9)	1.4 (1.0–2.0)	1.4 (1.1–1.7)
Two or more comorbidities	1.1 (0.9–1.4)	1.1 (0.9–1.4)	1.1 (0.9–1.4)	1.5 (1.1–1.9)	1.4 (1.1–1.8)	1.5 (1.2–1.9)	1.5 (1.1–2.2)	1.4 (1.1–1.8)
**Sex**								
Female	Ref.	Ref.	Ref.	Ref.	Ref.	Ref.	Ref.	Ref.
Male	0.8 (0.7–1.1)	0.8 (0.6–1.0)	0.8 (0.6–1.0)	0.9 (0.6–1.2)	0.9 (0.6–1.2)	0.8 (0.6–1.1)	0.8 (0.5–1.2)	0.8 (0.6–1.2)
**Age at diagnosis in years**								
< 60	Ref.	Ref.	Ref.	Ref.	Ref.	Ref.	Ref.	Ref.
60–69	1.1 (0.8–1.4)	1.1 (0.8–1.4)	1.1 (0.8–1.4)	1.0 (0.8–1.4)	1.0 (0.8–1.4)	0.9 (0.7–1.3)	1.0 (0.7–1.5)	1.0 (0.8–1.3)
70–79	1.4 (1.1–1.8)	1.4 (1.1–1.8)	1.4 (1.1–1.8)	1.3 (1.0–1.7)	1.2 (0.9–1.6)	1.3 (1.0–1.7)	1.1 (0.7–1.6)	1.4 (1.1–1.9)
≥ 80	2.7 (2.1–3.5)	2.1 (1.6–2.8)	2.1 (1.6–2.7)	2.0 (1.5–2.7)	1.7 (1.2–2.3)	1.9 (1.4–2.6)	2.1 (1.3–3.4)	2.1 (1.6–2.8)
**Histology**								
ADE		Ref.	Ref.	Ref.	Ref.	Ref.	Ref.	Ref.
Others		1.9 (1.5–2.4)	1.9 (1.5–2.3)	1.7 (1.3–2.1)	1.6 (1.3–2.0)	1.8 (1.4–2.2)	1.5 (1.0–2.2)	1.8 (1.4–2.2)
SCLC		1.1 (0.8–1.5)	1.1 (0.8–1.4)	0.9 (0.7–1.3)	0.7 (0.5–1.0)	0.8 (0.6–1.1)	0.7 (0.4–1.0)	0.9 (0.6–1.2)
SQUA		0.7 (0.6–1.0)	0.8 (0.6–1.0)	0.7 (0.5–0.9)	0.8 (0.6–1.0)	1.0 (0.7–1.3)	0.9 (0.6–1.3)	1.0 (0.7–1.2)
**Province**								
Girona			Ref.	Ref.	Ref.	Ref.	Ref.	Ref.
Granada			1.3 (1.1–1.6)	1.5 (1.3–1.9)	1.5 (1.2–1.8)	1.4 (1.2–1.7)	1.8 (1.4–2.4)	1.4 (1.1–1.7)
**Smoking status at diagnosis**								
Never smoked				Ref.	Ref.	Ref.	Ref.	Ref.
Previous smoker				1.4 (1.0–2.1)	1.5 (1.1–2.2)	1.5 (1.0–2.1)	2.0 (1.0–3.8)	1.6 (1.1–2.3)
Current smoker				1.7 (1.2–2.5)	1.7 (1.2–2.4)	1.6 (1.1–2.3)	2.1 (1.1–3.9)	1.5 (1.0–2.1)
**Surgery**								
Yes					Ref.	Ref.	Ref.	Ref.
No					16.0 (8.0–32.3)	9.4 (4.6–19.1)	6.2 (3.0–13.1)	10.2 (5.0–20.8)
**TNM stage**								
I-III (No-metastasis)						Ref.	Ref.	Ref.
IV (Metastasis)						2.4 (1.9–3.0)	2.5 (1.8–3.5)	2.3 (1.9–2.8)
**Body Mass Index (kg/m**^**2**^**)**								
< 24.9							Ref.	Ref.
25.0–29.9							1.0 (0.7–1.3)	0.9 (0.6–1.2)
≥ 30							0.8 (0.6–1.2)	0.7 (0.5–1.1)

**HR**: hazard ratio; **CI**: confidence interval

**Histology: ADE**: adenocarcinoma; **SCLC**: small cell lung cancer; **SQUA**: Squamous carcinoma; **Others**: non-small cell lung cancer, large cell lung cancer, neuroendocrine lung cancer, and unspecified lung cancer

**Model 1**: adjusted for sex and age;

**Model 2**: adjusted for sex, age and histology;

**Model 3**: adjusted for sex, age, histology and province of residence;

**Model 4**: adjusted for sex, age, histology, province of residence and smoking status;

**Model 5:** adjusted for sex, age, histology, province of residence, smoking status, and cancer surgery;

**Model 6:** adjusted for sex, age, histology, province of residence, smoking status, cancer surgery, and TNM stage;

**Model 7:** adjusted for sex, age, histology, province of residence, smoking status, cancer surgery, TNM stage, and BMI;

**Model 8:** model 7 with imputed BMI, TNM stage, surgery, and smoking status

## Discussion

This population-based cohort study revealed that both a single comorbidity and multimorbidity were consistent and independent prognostic factors of short-term mortality among lung cancer patients in Spain. Six months after diagnosis, lung cancer patients with one or multiple comorbidities had a 40% higher risk of all-cause mortality than those without comorbidities, after adjusting for age, sex, histology, smoking status, province of residence, performed surgery, BMI, and tumour stage. We found high prevalence of comorbidity in lung cancer patients, especially among the elderly; men; those diagnosed with advanced-stage; smokers and obese patients.

We confirmed findings from other population-based studies that found comorbidity to be prognostically relevant and associated with lung cancer mortality, after controlling for relevant confounders, such as age, sex, or stage at diagnosis [27–30]. This association was not replicated in other, mainly smaller single-centre studies [12–14]. The discrepancy may be because the latter were single-centre studies that failed to take into account relevant confounders, [12–14] or dated population-based studies where common comorbidities, such as cardiovascular diseases, were underreported [15]. Sandfeld-Paulsen et al. [31] argue that the association between comorbidity and lung cancer mortality can be detected only by using register-based data, including reliable information on all cancer patients in a defined region and time period. However, the main disadvantage of population-based studies is that they often fail to consider relevant lifestyle and behavioural factors, such as smoking status. To the best of our knowledge, our study is unique in revealing that this association remains stable after controlling for relevant lifestyle factors, such as obesity and smoking status. Furthermore, the estimated mortality risk in our study was in line with a review of studies, which concluded that mortality in lung cancer patients was between 1.1 to 1.5 times higher for patients with than those without comorbidity [17].

We did not find that the mortality risk was higher with an increasing number of chronic conditions, since the presence of either comorbidity or multimorbidity had comparable impact on lung cancer mortality. Results from previous studies are conflicting; while some studies found this gradient, [29, 32] others did not [14, 33]. This may be partly attributable to patients’ clinical characteristics. Studies including early stage lung cancer patients were more likely to report that multimorbidity contributed to increased mortality, while those including patients with advanced-stage cancer did not find multimorbidity had any important prognostic value [9]. For patients with early-stage lung cancer or any potentially curable cancer, such as early-stage breast or prostate cancer, the presence and number of comorbid conditions may be more likely to predict their mortality risk [9, 10]. On the other hand, patients diagnosed with advanced-stage disease or more aggressive cancers with poor prognosis, such as lung cancer, are more likely to die from their cancer regardless of other concomitant disease.

We found that the impact of comorbidity on lung cancer mortality was independent of cancer stage or patients’ age, but these factors may be prognostically complementary to comorbidity status [30]. Elderly patients with metastasis had a two times higher mortality risk than younger patients without metastasis, confirming findings from previous studies [27, 34]. We argue that advanced age is a prognostic factor of mortality, because the elderly tend to receive less active lung cancer treatment, including less chemotherapy, radiotherapy or surgery than younger patients [35].

Although surgical resection remains the main and most effective lung cancer treatment, it is only indicated in early stage tumours [36]. Our study included 16% of patients with stage I and II tumours, with the matching 16% of performed surgeries. The probability of successful surgery is further reduced with advanced age and the presence of comorbidities, mainly due to the expected higher incidence of postoperative complications [15, 37]. Evidence suggests that between 24 and 70% of cancer patients with comorbidity are not treated according to guidelines [17]. We did not have reliable information whether surgery was performed with curative intent. Even if a small number of patients had surgery, we found that it was highly protective of mortality risk. Therefore, we argue that in most cases surgery was performed with curative intent.

Sex did not affect the short-term mortality, when relevant confounders were considered, although women had lower mortality risk than men. Consistent with our findings, other studies found that lung cancer mortality trends were higher among women than men, but mortality rates were still higher among Spanish men [38, 39]. An increasing trend of tobacco use among women and the decreasing trend among men may contribute to this [38]. Granada had consistently higher overall short-term mortality than the wealthier Northern region of Girona, which confirms that social inequalities may play an important role in lung cancer mortality [40].

Comorbidity remains more prevalent among patients diagnosed with lung than other cancer types [10, 17, 41, 42]. A possible explanation may be found in lifestyle factors contributing to lung cancer, especially smoking and obesity - the main risk factors for many chronic conditions [17, 29]. An estimated 26–81% of lung cancer patients have at least one other chronic medical condition i.e., comorbidity, consistent with our results [17]. A large population-based cohort study, done in Canada, found that almost all (91%) people diagnosed with different types of cancer had other chronic medica conditions; and almost a quarter (23%) of them had five or more co-occurring conditions [42]. They found that lung cancer patients had among the highest prevalence of multimorbidity than patients diagnosed with 15 other types of cancer, and early death most commonly occurred among the lung cancer patients. Different from Koné and Scharf, however, is that we did not find that mortality is increasing with higher number of chronic medical conditions. This may be because instead of focusing on patients diagnosed with a range of different cancers, we analysed lung cancer patients only, who have worse prognosis and higher mortality than most other cancer patients. Moreover, we adjusted our analysis for lifestyle factors, such as smoking and obesity status, and clinical factors, such as tumour histology and surgery, which may contribute to this discrepancy.

We confirmed high smoking prevalence among lung cancer patients in Spain [43]. Smoking contributes to over 80% of lung cancers in high-income countries, and, therefore, preventive strategies require strict tobacco control [1]. The most common comorbid conditions were age- and tobacco-related illnesses, such as respiratory (e.g., COPD) and cardiovascular (e.g., heart failure) diseases, consistent with earlier studies [15, 36]. Significant overlap occurs between symptoms of these diseases and lung cancer, including cough, dyspnea and chest tightness [44]. Another common comorbidity, unrelated to tobacco use, was diabetes, sharing the same risk factors with lung cancer, such as age, diet and smoking [36, 45]. Our findings highlight the most prevalent comorbidities among lung cancer patients and the pattern of correlations between the most common comorbidities. This highlights the need for further research to better understand the relationships between these chronic conditions and how they might interact. For example, are patients with comorbidities more likely to be diagnosed with lung cancer early because of their frequent medical appointments, or they are more likely to have delayed diagnosis because the symptoms of their comorbid diseases are masking early signs of lung cancer? Future studies should also explore whether these common comorbidities have an additive or a multiplicative effect on lung cancer mortality.

The majority of patients in our study had advanced-stage cancer, probably because early lung cancer symptoms are hard to recognise or they overlap with other diseases, such as COPD, causing delays in diagnosis [44]. A national lung cancer awareness campaign was introduced in England, following reports that early symptoms, such as “persistent cough or hoarseness” were least frequently recognised by the public, especially among the elderly, males and the socioeconomically disadvantaged people, who also reported most barriers to seeking medical help [46]. The campaign achieved an increase in the number of medical appointments and diagnostic tests, as well as a shift towards an early-stage lung cancer diagnosis, when outcomes are more favourable [47, 48]. There are currently no national campaigns in Spain aimed at raising awareness about early symptoms of lung cancer and encouraging early diagnosis. Introducing such campaigns may have long-term benefits for lung cancer patients in Spain.

This is the first high-resolution study addressing the effect of comorbidity on short-term overall mortality among lung cancer patients in Spain. Accessing patients’ EHRs allowed us to enrich data from two population-based cancer registries addressing lifestyle factors, such as smoking and BMI, not often assessed in population-based studies. We used multiple imputations to address missing values, performing analyses with both observed and imputed values. We used a relatively reliable comorbidity measure, reducing the possibility to misclassify the comorbid conditions, but some residual bias is possible due to unmeasured comorbidities.

This study has limitations. First, data were collected during 2011 and 2012, because the data collection was part of the European High Resolution studies and this was the most complete population cohort available. Although this must be considered when interpreting the results, it is unlikely that the distribution of lung cancer incidence and main comorbidities would be significantly different in the more recent years. Second, we were unable to perform analysis including the malnourished patients (i.e., BMI category < 18.5 kg/m2) due to a small number of cases in this category (*N* = 22). Future studies should explore the role of malnutrition, especially among the elderly, as it may contribute to adverse health outcomes, such as a more advanced neoplastic disease or COPD. Third, the RCS is a simple measurement, created to compare comorbidity in patients planned for a surgical intervention. Using this score can simplify the data collection from digital medical records, but some conditions may be excluded, such as psychiatric diseases, other neoplasms, hypertension, non-COPD chronic respiratory diseases or autoimmune diseases. Therefore, the presence of different comorbidities may be underrepresented, and its impact on short-term lung cancer mortality may be even higher than we estimated in this study. Fourth, the prevalence of comorbidities might not be applicable to other regions or countries. However, the clustering of chronic conditions is likely to have a synergistic effect on health outcomes, regardless of geographical coordinates. Finally, we did not have information on other lifestyle factors, such as exercise, diet, alcohol consumption, the onset and duration of smoking or detailed socio-demographic descriptors, such as patients’ income. Future studies should investigate these factors and include data from all population-based cancer registries in Spain.

## Conclusion

The presence of comorbid diseases, rather than the number of comorbidities, is associated with an increasing risk of short-term lung cancer mortality in Spain. Developing national or local campaigns focused on rising symptom awareness may encourage early lung cancer diagnosis. Additional efforts are required to introduce targeted preventive interventions, such as more rigorous smoking cessation interventions, which may decrease the incidence of lung cancers combined with other comorbid diseases. The development of more personalised healthcare guidelines is needed to address the complex treatment management of lung cancer patients with comorbidity in Spain.

## Supplementary Information

**Additional file 1 Supplementary Table 1.** Histology group classification. **Supplementary Table 2.** Comorbidities and their diagnostic ICD-10 codes. **Supplementary Table 3.** Comorbidity status, vital status at six-months, sociodemographic characteristics: smoking status, province of residence, BMI, cancer surgery, morphology and TNM stage among lung cancer patients diagnosed between 2010 and 2012, in two population-based Spanish cancer registries: Girona and Granada **(***n* = 1259 lung cancer patients and 581 deaths at six-months after cancer diagnosis). **Supplementary Table 4.** Top 5 most frequent multimorbidity patterns with 2 or 3 comorbidities and 6-month mortality risk among lung cancer patients diagnosed between 2011 and 2012, in two population-based Spanish cancer registries: Girona and Granada (*n* = 1259 and 581 deaths at six-months after cancer diagnosis).

**Additional file 2 Supplementary Table 5.** Short-term (six-months) comorbidity status mortality risk adjusted for sex, age, province of residence, smoking status, cancer surgery, histology, TNM stage, and BMI among lung cancer patients in Girona and Granada, Spain in 2011 (n = 1259 lung cancer patients and 581 deaths at six-months after cancer diagnosis).

## Data Availability

The data that support the findings of this study are available from the Regional Government of Andalusia and the Andalusian Health Department, but restrictions apply to the availability of these data, which is often the case with cancer registry data, and so are not publicly available. The Regional Government of Andalusia and the Andalusian Health Department should be contacted to access the raw data from the present study.
