# Supplementary material for: The role of multimorbidity in short-term mortality of lung cancer patients in Spain: a population-based cohort study
Source: BMC Cancer. 2021 Sep 24;21:1048. doi: 10.1186/s12885-021-08801-9 (PMC8461961; doi:10.1186/s12885-021-08801-9)
Supplement: Supplementary file 1 — Additional file 1 Supplementary Table 1. Histology group classification. Supplementary Table 2. Comorbidities and their diagnostic ICD-10 codes. Supplementary Table 3. Comorbidity status, vital status at six-months, sociodemographic characteristics: smoking status, province of residence, BMI, cancer surgery, morphology and TNM stage among lung cancer patients diagnosed between 2010 and 2012, in two population-based Spanish cancer registries: Girona and Granada (n = 1259 lung cancer patients and 581 deaths at six-months after cancer diagnosis). Supplementary Table 4. Top 5 most frequent multimorbidity patterns with 2 or 3 comorbidities and 6-month mortality risk among lung cancer patients diagnosed between 2011 and 2012, in two population-based Spanish cancer registries: Girona and Granada (n = 1259 and 581 deaths at six-months after cancer diagnosis). [file 12885_2021_8801_MOESM1_ESM.doc]

**Supplementary Tables (n=4)**

**Authors:** Maja Niksic, Daniel Redondo-Sanchez, Yoe-Ling Chang, Miguel Rodriguez-Barranco, Jose Exposito-Hernandez, Rafael Marcos-Gragera, Ester Oliva-Poch, Joaquim Bosch-Barrera, Maria-Jose Sanchez, Miguel Angel Luque-Fernandez

**Title:** The role of multimorbidity in short-term mortality of lung cancer patients in Spain: A population-based cohort study

**Supplementary Table 1.** Histology group classification

| **Histology subgroup** | **ICD-O-3 histological (morphological) code** |
| --- | --- |
| Adenocarcinoma (ADE) | 8140; 8200; 8211; 8260; 8230; 8255; 8310; 8323; 8430; 8480; 8481; 8490; 8550; 8560; 8570 |
| Others | Unspecified carcinoma - 8000; 8001; 8010; 8031; 8020; 8021; 8022;  Non-small cell lung cancer - 8046; 8250-8252; 8253;  Large cell carcinoma - 8012  Other - 8033; 8082; 8800; 8815; 8980; 9140; 9220; 9530;  Neuroendocrine Carcinoma - 8013; 8240; 8244; 8246; 8249; 8574; |
| Small cell lung cancer (SCLC) | 8041; 8042; 8043; 8044; 8045; |
| Squamous carcinoma (SQUA) | 8052; 8070; 8071; 8072; 8073; 8074; 8075; 8076; 8083; 8084; 8123; |

**ICD-O-3**: International Classification of Diseases for Oncology, 3^rd^ edition

**Supplementary Table 2.** Comorbidities and their diagnostic ICD-10 codes

|  | **Comorbidity** |  | **ICD-10** |  |
| --- | --- | --- | --- | --- |
|  | Myocardial infarction |  | I21.x, I22.x, I25.2 |  |
|  | Congestive heart failure |  | I11.0, I13.0, I13.2, I25.5, I42.0, I42.5–I42.9, I43.x, I50.x, P29.0 |  |
|  | Peripheral vascular disease |  | I70.x, I71.x, I73.1, I73.8, I73.9, I77.1, I79.0, I79.2, K55.1, K55.8, K55.9, Z95.8, Z95.9 |  |
|  | Cerebrovascular disease |  | G45.x, G46.x, H34.0, I60.x–I69.x |  |
|  | Dementia |  | F00.x–F03.x, F05.1, G30.x, G31.1 |  |
|  | Chronic obstructive pulmonary disease |  | I27.9, J40.x–J47.x, J60.x–J67.x, J68.4, J70.1, J70.3 |  |
|  | Rheumatic disease |  | M05.x, M06.x, M31.5, M32.x–M34.x, M35.1, M35.3, M36.0 |  |
|  | Liver disease |  | B18.x, K70.0–K70.3, K70.9, K71.3–K71.5, K71.7, K73.x, K74.x, K76.0, K76.2–K76.4, K76.8, K76.9, Z94.4, K71.1, K72.1, K72.9, K76.5, K76.6, K76.7, I85.0, I85.9, I86.4, I98.2, K70.4, |  |
|  | Diabetes |  | E10.0, E10.1, E10.6, E10.7, E10.8, E10.9, E11.x, E12.x, E13.x, E14.x |  |
|  | Hemiplegia or paraplegia |  | G04.1, G11.4, G80.1, G80.2, G81.x, G82.x, G83.0–G83.4, G83.9 |  |
|  | Renal disease |  | I12.0, I13.1, N03.2–N03.7, N05.2–N05.7, N18.x, N19.x, N25.0, Z49.0–Z49.2, Z94.0, Z99.2 |  |
|  | AIDS/HIV |  | B20.x–B22.x, B24.x |  |

**ICD-10**: International Classification of Diseases, 10th Revision

**Supplementary Table 3.** Comorbidity status, vital status at six-months, sociodemographic characteristics: smoking status, province of residence, BMI, cancer surgery, morphology and TNM stage among lung cancer patients diagnosed between 2010 and 2012, in two population-based Spanish cancer registries: Girona and Granada **(**n = 1,259 lung cancer patients and 581 deaths at six-months after cancer diagnosis).

| **Variable** | **N (%)** |
| --- | --- |
| **Comorbidity status** |  |
| No comorbidity | 486 (38.6) |
| Comorbidity | 353 (28.0) |
| Multimorbidity | 420 (33.4) |
| **Comorbidity** |  |
| COPD | 438 (36.6) |
| Diabetes | 248 (20.7) |
| Congestive heart failure | 200 (16.7) |
| Peripheral vascular disease | 114 (9.5) |
| Myocardial infarction | 107 (9.0) |
| Cerebrovascular disease | 88 (7.4) |
| Renal disease | 68 (5.7) |
| Rheumatic disease | 55 (4.6) |
| Liver disease | 53 (4.4) |
| Dementia | 37 (3.1) |
| AIDS/HIV | 9 (0.8) |
| Hemiplegia | 2 (0.2) |
| **Vital status at 6 months** |  |
| Alive | 678 (53.9) |
| Dead | 581 (46.1) |
| **Age at diagnosis, years** |  |
| <60 | 304 (24.1) |
| 60–69 | 335 (26.6) |
| 70–79 | 387 (30.7) |
| ≥80 | 233 (18.5) |
| **Sex** |  |
| Male | 1050 (83.4) |
| Female | 209 (16.6) |
| **Smoking status** |  |
| Current smoker | 453 (41.3) |
| Former smoker | 501 (45.7) |
| Never smoked | 142 (12.9) |
| **Province** |  |
| Girona | 499 (39.6) |
| Granada | 760 (60.3) |
| **Body Mass Index (kg/m^2^)** |  |
| <24.9 | 310 (42.1) |
| 25.0–29.9 | 276 (37.5) |
| ≥30 | 151 (20.5) |
| **Surgery** |  |
| No | 1,012 (83.4) |
| Yes | 202 (16.6) |
| **Histology** |  |
| ADE | 382 (30.3) |
| Others | 378 (30.0) |
| SCLC | 165 (13.1) |
| SQUA | 334 (26.5) |
| **TNM stage** |  |
| I | 5 (0.4) |
| II | 191 (15.9) |
| III | 349 (29.0) |
| IV | 659 (54.7) |

**Comorbidity:** One comorbidity, **Multimorbidity:** Two or more comorbidities, **Histology:** ADE: adenocarcinoma; SCLC: small cell lung cancer; SQUA: Squamous carcinoma; Others: non-small cell lung cancer, large cell lung cancer, neuroendocrine lung cancer, and unspecified lung cancer.

**Missing values:** Chronic obstructive pulmonary disease (COPD) n (%) = 64 (5.1%), Diabetes n (%) = 64 (5.1%), Congestive heart failure n (%) = 64 (5.1%), Peripheral vascular disease n (%) = 65 (5.2%), Myocardial infarction n (%) = 64 (5.1%), Cerebrovascular disease infarction n (%) = 64 (5.1%), Renal disease n (%) = 64 (5.1%), Rheumatic disease n (%) = 64 (5.1%), Liver disease n (%) = 64 (5.1%), Dementia n (%) = 64 (5.1%), AIDS/HIV n (%) = 64 (5.1%), Hemiplegia n (%) = 64 (5.1%), BMI n(%) = 523 (41.5%) , Smoking status n(%) = 163 (12.9), TNM stage n (%) = 55 (4.4%), Surgery n(%) = 45 (3.6)

**Supplementary Table 4.** Top 5 most frequent multimorbidity patterns with 2 or 3 comorbidities and 6-month mortality risk among lung cancer patients diagnosed between 2011 and 2012, in two population-based Spanish cancer registries: Girona and Granada (n = 1,259 and 581 deaths at six-months after cancer diagnosis).

| **Top 5 multimorbidity patterns** (2 comorbidities) | **N (%)** |
| --- | --- |
| Chronic obstructive pulmonary disease + Diabetes | 110 (8.7%) |
| Chronic obstructive pulmonary disease + Congestive heart failure | 99 (7.9%) |
| Congestive heart failure + Diabetes | 70 (5.6%) |
| Chronic obstructive pulmonary disease + Myocardial infarction | 54 (4.3%) |
| Chronic obstructive pulmonary disease + Peripheral vascular disease | 54 (4.3%) |
| **Top 5 multimorbidity patterns** (3 comorbidities) | **N (%)** |
| Chronic obstructive pulmonary disease + Congestive heart failure + Diabetes | 37 (2.9%) |
| Chronic obstructive pulmonary disease + Myocardial infarction + Diabetes | 20 (1.6%) |
| Chronic obstructive pulmonary disease + Myocardial infarction + Congestive heart failure | 17 (1.4%) |
| Chronic obstructive pulmonary disease + Congestive heart failure + Renal disease | 16 (1.3%) |
| Chronic obstructive pulmonary disease + Congestive heart failure + Peripheral vascular disease | 14 (1.1%) |
