# Supplementary material for: The role of multimorbidity in short-term mortality of lung cancer patients in Spain: a population-based cohort study
Source: BMC Cancer. 2021 Sep 24;21:1048. doi: 10.1186/s12885-021-08801-9 (PMC8461961; doi:10.1186/s12885-021-08801-9)
Supplement: Supplementary file 2 — Additional file 2 Supplementary Table 5. Short-term (six-months) comorbidity status mortality risk adjusted for sex, age, province of residence, smoking status, cancer surgery, histology, TNM stage, and BMI among lung cancer patients in Girona and Granada, Spain in 2011 (n = 1259 lung cancer patients and 581 deaths at six-months after cancer diagnosis). [file 12885_2021_8801_MOESM2_ESM.doc]

**Supplementary Table 5.** Short-term (six-months) comorbidity status mortality risk adjusted for sex, age, province of residence, smoking status, cancer surgery, histology, TNM stage, and BMI among lung cancer patients in Girona and Granada, Spain in 2011 (n = 1,259 lung cancer patients and 581 deaths at six-months after cancer diagnosis).

| **Variables** | **N (%)** | **HR (95%CI)** | | |
| --- | --- | --- | --- | --- |
| No comorbidity | 486 (38.6) | | Ref. |  |
| One comorbidity | 353 (28.0) | | 1.5 (1.2–1.9) |  |
| Two comorbidities | 270 (21.5) | | 1.5 (1.2–2.0) |  |
| Three or more comorbidities | 150 (11.9) | | 1.4 (1.0–1.9) |  |

**HR**: hazard ratio; **CI**: confidence interval.

**A**djusted for sex, age, histology, province of residence, smoking status, cancer surgery, TNM stage, and BMI
